# Osteosarcoma immune prognostic index can indicate the nature of indeterminate pulmonary nodules and predict the metachronous metastasis in osteosarcoma patients

**DOI:** 10.3389/fonc.2022.952228

**Published:** 2022-07-22

**Authors:** Xuanhong He, Minxun Lu, Xin Hu, Longqing Li, Chang Zou, Yi Luo, Yong Zhou, Li Min, Chongqi Tu

**Affiliations:** Department of Orthopedics, Orthopaedic Research Institute, West China Hospital, Sichuan University, Chengdu, China

**Keywords:** osteosarcoma, inflammation, osteosarcoma immune prognostic index, metastatic predictive markers, indeterminate pulmonary nodules

## Abstract

**Purpose:**

The relationship between indeterminate pulmonary nodules (IPNs) and metastasis is difficult to determine. We expect to explore a predictive model that can assist in indicating the nature of IPNs, as well as predicting the probability of metachronous metastasis in osteosarcoma patients.

**Patients and methods:**

We conducted a retrospective study including 184 osteosarcoma patients at West China Hospital from January 2016 to January 2021. Hematological markers and clinical features of osteosarcoma patients were collected and analyzed.

**Results:**

In this study, we constructed an osteosarcoma immune prognostic index (OIPI) based on the lung immune prognostic index (LIPI). Compared to other hematological markers and clinical features, OIPI had a better ability to predict metastasis. OIPI divided 184 patients into four groups, with the no-OIPI group (34 patients), the light-OIPI group (35 patients), the moderate-OIPI group (75 patients), and the severe-OIPI group (40 patients) (P < 0.0001). Subgroup analysis showed that the OIPI could have a stable predictive effect in both the no-nodule group and the IPN group. Spearman’s rank correlation test and Kruskal–Wallis test demonstrated that the OIPI was related to metastatic site and metastatic time, respectively. In addition, patients with IPNs in high-OIPI (moderate and severe) groups were more likely to develop metastasis than those in low-OIPI (none and light) groups. Furthermore, the combination of OIPI with IPNs can more accurately identify patients with metastasis, in which the high-OIPI group had a higher metastasis rate, and the severe-OIPI group tended to develop metastasis earlier than the no-OIPI group. Finally, we constructed an OIPI-based nomogram to predict 3- and 5-year metastasis rates. This nomogram could bring net benefits for more patients according to the decision curve analysis and clinical impact curve.

**Conclusion:**

This study is the first to assist chest CT in diagnosing the nature of IPNs in osteosarcoma based on hematological markers. Our findings suggested that the OIPI was superior to other hematological markers and that OIPI can act as an auxiliary tool to determine the malignant transformation tendency of IPNs. The combination of OIPI with IPNs can further improve the metastatic predictive ability in osteosarcoma patients.

## Introduction

Osteosarcoma is the most common primary bone malignancy primarily affecting children, adolescents, and the elderly (1). Current standard treatment for primary osteosarcoma includes neoadjuvant chemotherapy, wide surgical resection and adjuvant chemotherapy (1, 2). As comprehensive treatment advances, the 5-year overall survival (OS) rate improves to 60%–70%, while it decreases to 20%–30% when metastasis occurs (3). Metastasis remains the biggest obstacle to the clinical outcome of osteosarcoma (3, 4). Almost all osteosarcoma patients have subclinical micrometastatic disease at the time of initial diagnosis; however, metastatic status can be detected in only 20% of patients (5, 6). Patients with subclinical micrometastases frequently develop metastatic disease during follow-up, mainly leading to clinical treatment failure and a fatal clinical course (7–9).

The lung is the main metastatic site in patients with osteosarcoma (3, 10). An accurate evaluation of the lung metastatic status is essential. In the early stages of metastasis, lung metastasis always presents as micrometastasis; these micrometastases are difficult to distinguish from other benign nodules (3, 11, 12). Those benign and malignant undetermined pulmonary nodules are called indeterminate pulmonary nodules (IPNs); they are defined as non-calcified nodules with a maximum diameter <10 mm (13–15). With the application of fine-section computed tomography (CT), more suspicious pulmonary nodules are detected, bringing new challenges for accurate identification of pulmonary metastasis status in osteosarcoma patients (16). Since IPNs are not specific in cancer patients, the identification of their nature is difficult or even not feasible (11, 12, 16, 17). Due to the size of IPNs, needle biopsy is usually not feasible, and as an invasive test, biopsy may be excessive (17, 18). Clinically, radiologists often estimate the probability of lung metastasis with nodule size, margin, presence of calcification, and nodule amount (19–21). However, available studies suggest that none of these features could adequately distinguish malignancy from benign lesions (11). Therefore, more markers need to be included to assist in the judgment of the nature of IPNs.

Timely determination of the nature of IPNs and accurate development of individualized treatment decisions could improve the prognosis of osteosarcoma patients (3, 11, 17). In recent years, researchers have tried to determine the nature of IPNs by cell-free DNA (cfDNA), microRNA (miRNA), and circulating tumor cells (CTCs) (22). These specimens have the advantage of being non-invasive and reproducible (22). However, due to the limitation of sensitivity, specificity, and high cost, these biomarkers cannot be applied in clinical practice. In addition, an increasing number of studies have shown that hematological markers (such as the neutrophil–lymphocyte ratio (NLR), platelet–lymphocyte ratio (PLR), lymphocyte–monocyte ratio (LMR), and serum lactate dehydrogenase (LDH)) are associated with the prognosis of various cancers, including osteosarcoma (23–28). More surprisingly, some hematological markers, such as the lung immune prognostic index (LIPI), can predict the response to immunotherapy by reflecting the proinflammatory status (29–33). Therefore, it is reasonable to speculate that these hematological markers could assist in judging the nature of IPNs.

To the best of our knowledge, there have been no studies applying hematological markers to assist in the judgment of the nature of IPNs in osteosarcoma. Therefore, the main purpose of this study was to evaluate the value of classical hematological markers such as NLR, PLR, and LMR and the combination of hematological markers of LIPI in the judgment of the nature of IPNs. In addition, we modified the construction of the OIPI based on the LIPI and assessed its ability to predict the nature of IPNs.

## Patients and methods

### Patients

After obtaining institutional review board approval, we retrospectively reviewed the clinical data of osteosarcoma patients from January 2016 to January 2021 in the database of the Musculoskeletal Tumor Center of West China Hospital. The inclusion criteria were as follows (1): patients with high-grade osteosarcoma confirmed by histopathology; 2) patients with complete hematological test results and staging chest CT prior to the neoadjuvant chemotherapy; and 3) patients who received standard treatment at West China Hospital. The exclusion criteria were as follows: 1) patients who had received neoadjuvant chemotherapy before their first-time consultancy in our hospital; 2) patients with hematological diseases and other malignancies (3); patients who did not receive staging chest CT during the follow-up; and (4) patients with distal metastasis at the time of diagnosis. Finally, a total of 184 patients were included in our study. Metastasis-free survival (MFS) was calculated from the date of diagnosis to the date of metastasis or last follow-up. Each patient was regularly followed up until death or January 2022. All the patients obeyed the follow-up rules: reexamination every 3 months within 1 year after surgery; every 4 months for 1–2 years after surgery; every 5 months for 2–3 years after surgery; twice a year for 3–5 years after surgery; and yearly after 5 years postoperatively.

### Data collection and processing

Leukocyte count (Leut#), neutrophil count (Neut#), lymphocyte count (LYMPH#), monocyte count (MONO#), platelet count (PLT), lactate dehydrogenase (LDH), hydroxybutyrate dehydrogenase (HBDH), creatine kinase (CK), and alkaline phosphatase (ALP) were extracted from the blood routine of the 184 patients prior to neoadjuvant chemotherapy. The formulas for calculating NLR, PLR, LMR, and dNLR are as follows: NLR = Neut#/LYMPH#, PLR = PLT/LYMPH#, LMR = LYMPH#/MONO#, and dNLR= Neut#/(Leut#-Neut#). In addition, age, gender, tumor site, pathologic fracture status, and tumor metastasis status were collected from the patients’ medical records. In the overall cohort, the optimal cutoff value for each hematological marker was calculated based on the time-dependent receiver operating curve (ROC) and converted into a binary variable according to the cutoff value.

### Establishment and validation of the OIPI

Referring to the development of the LIPI, we established the prognostic model OIPI by combining the hematological indexes with a higher area under curve (AUC) in the ROC curves according to our results. Then, we compared the prognostic predictive effect of the OIPI with that of other hematological factors and clinical characteristics by time-dependent ROC. To verify whether the OIPI is an independent risk factor for metastasis in osteosarcoma patients, we conducted univariate and multivariate analyses. Significant factors in univariate analyses were then subjected to multivariate analyses to determine independent risk factors for metastasis. Furthermore, the association between the OIPI and metastatic sites or metachronous metastasis time was also explored by Spearman’s rank correlation analysis, Kaplan–Meier survival analysis, and Kruskal–Wallis test.

### Identification of IPNs

Computed tomography scans and reports were reviewed by at least two radiologists. Rissing et al. (13), defined IPNs as non-calcified nodules <10 mm in maximal diameter; according to this, all osteosarcoma patients were classified as no-nodule and IPNs at the time of diagnosis. All the patients received staging chest CT during the follow-up. Patients were considered to have pulmonary metastases when the following first occurred: maximum diameter of a pulmonary nodule increased by at least 25%, subsequent appearance of new pulmonary nodules on chest CT during follow-up, nodules were pathologically diagnosed as metastases, the date the treating oncologist documented the presence of pulmonary metastasis, or the date the oncologist documented the occurrence of pulmonary metastases.

### Construction and validation of the OIPI-based nomogram

After a multistep screening process, an osteosarcoma metachronous metastasis nomogram was constructed combining the OIPI and clinical features. For each osteosarcoma patient, the total point was equal to the sum of the points of all metastasis prediction factors. The relationships between the total points and the probability of MFS are shown at the bottom of the nomogram. We evaluated the discrimination ability and accuracy of the nomogram by Harrell’s concordance index and calibration curve, respectively. In the calibration curve, the diagonal acts as a reference line and represents the best prediction. Decision curve analysis (DCA) was used to evaluate the clinical application of the nomogram by estimating the net benefits at different threshold probabilities. The clinical impact curve was also drawn to predict reduction intervention probability per 100 patients.

### Statistical analysis

The Kolmogorov–Smirnov test was used to assess whether continuous variables were normally distributed, and the Mann–Whitney U test or Spearman correlation analysis was used to assess differences between continuous variables according to the results. Categorical variables were evaluated using the chi-square test and Fisher’s exact test based on the number of individuals in each group. All statistical analyses were conducted using R software, version 4.1.0 (Institute for Statistics and Mathematics, Vienna, Austria). P-values < 0.05 were considered to indicate statistical significance.

## Results

### Patient characteristics and optimal cutoff values of hematological factors

The patients included in this study consisted of 107 men and 77 women. The age of the patients ranged from 7 to 67 years, with a mean age of 21 years. Tumors were mainly located at the extremities in 176 patients, and only eight patients had tumors affecting the extra-extremities. Pathological fracture at the time of diagnosis and metachronous metastasis were found in 19 and 64 patients respectively. The time to develop metachronous metastasis in the 64 patients ranged from 2 to 52 months with a mean time of 14.7 months. Among 184 osteosarcoma patients, the average MFS was 30.5 months. In addition, according to the initial chest CT, 67 of 184 patients were classified as having IPNs and 117 of 184 patients were classified as having no nodule. In 64 patients who developed metachronous metastasis, 32 patients had no nodule and 32 patients had IPNs (**Table 1**). The optimal cutoff values for hematological markers are also shown in **Supplementary Table 1** (NLR, PLR, LMR, LDH, dNLR, HBDH, CK, ALP).

**Table 1:** Characteristics of 184 osteosarcoma patients.

Characteristics	Patients, n (%)		P-value
	Non-metastasis(n = 120)	Metachronous metastasis(n = 64)	
Age (years)			
>22	35 (29.2%)	19 (29.7%)	1.000
≤22	85 (70.8%)	45 (70.3%)
Gender			
Male	54 (45.0%)	41 (64.1%)	0.273
Female	66 (55.0%)	23 (35.9%)
Tumor site			
Extremities	116 (96.7%)	60 (93.8%)	0.452
Non-extremities	4 (3.3%)	4 (6.2%)
Pathological fracture			
Yes	9 (7.5%)	10 (15.6%)	0.125
No	111 (92.5%)	54 (84.4%)
Chest CT			
No-nodule	85 (70.8%)	32 (50.0%)	0.006
IPNs	35 (29.2%)	32 (50.0%)
NLR			
Low	83 (69.2%)	28 (43.8%)	0.001
High	37 (30.8%)	36 (56.2%)
PLR			
Low	100 (83.3%)	42 (65.6%)	0.009
High	20 (16.7%)	22 (34.4%)
LMR			
Low	78 (65.0%)	48 (75.0%)	0.001
High	42 (35.0%)	16 (25.0%)
LDH			
Low	51 (42.5%)	13 (20.3%)	0.003
High	69 (57.5%)	51 (79.7%)
dNLR			
Low	85 (70.8%)	31 (48.4%)	0.004
High	35 (29.2%)	33 (51.6%)
HBDH			
Low	58 (48.3%)	9 (14.1%)	0.000
High	62 (51.7%)	55 (85.9%)
CK			
Low	104 (86.7%)	48 (75.0%)	0.065
High	16 (13.3%)	16 (25.0%)
ALP			
Low	67 (55.8%)	26 (40.6%)	0.063
High	53 (44.2%)	38 (59.4%)
LIPI			
Good	36 (30.0%)	6 (9.4%)	0.000
Intermediate	64 (53.3%)	32 (50.0%)
Poor	20 (16.7%)	26 (40.6%)
OIPI			
None	31 (25.8%)	3 (4.7%)	0.000
Light	27 (22.5%)	8 (12.5%)
Moderate	47 (39.2%)	28 (43.8%)
Poor	15 (12.5%)	25 (39.0%)

IPNs, indeterminate pulmonary nodules; NLR, neutrophil–lymphocyte ratio; PLR, platelet–lymphocyte ratio; LMR, lymphocyte–monocyte ratio; LDH, lactate dehydrogenase; dNLR, derived neutrophil to lymphocyte ratio; HBDH, hydroxybutyrate dehydrogenase; CK, creatine kinase; ALP, alkaline phosphatase; LIPI, lung immune prognostic index; OIPI, osteosarcoma immune prognostic index.

**Figure 1:**
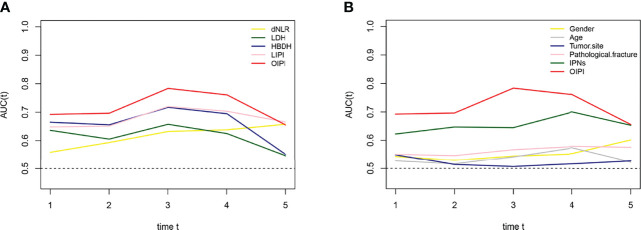
Comparison of different clinical biomarkers in predicting metachronous metastasis. **(A)** The difference in the predictive ability of different hematological markers is shown in the time-dependent ROC curve, in which a larger AUC value indicates a better metastatic predictive ability. **(B)** Difference in the predictive ability of different clinical features. dNLR, derived neutrophil-to-lymphocyte ratio; LDH, lactate dehydrogenase; HBDH, hydroxybutyrate dehydrogenase; LIPI, lung immune prognostic index; OIPI, osteosarcoma immune prognostic index; ROC, receiver operating curve; AUC, area under the curve; IPNs, indeterminate pulmonary nodules.

**Figure 2:**
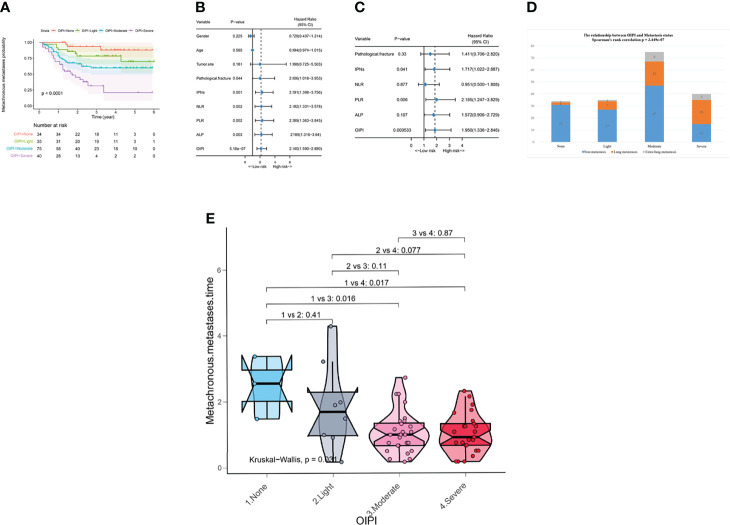
Effect of the OIPI in predicting metachronous metastasis, metastatic sites, and metastatic time in 184 patients. **(A)** OIPI divided 184 osteosarcoma patients into four groups. According to the logistic regression analysis, the differences between four OIPI groups in the metachronous metastasis probability were significant. **(B)** Forest plot showing the results of univariate Cox regression analysis of hematological markers and clinical features in 184 osteosarcoma patients. **(C)** Forest plot showing the results of multivariate Cox regression analysis of hematological markers and clinical features in 184 osteosarcoma patients. **(D)** Spearman’s rank correlation test demonstrated that OIPI was related to metastatic sites. **(E)** Kruskal–Wallis test demonstrated that there was a difference in metastatic time among four groups (P = 0.031). Patients in moderate and severe OIPI groups developed metachronous metastasis earlier than that of the no-OIPI group (P = 0.016 and P = 0.017, respectively). OIPI, osteosarcoma immune prognostic index; IPNs, indeterminate pulmonary nodules; NLR, neutrophil–lymphocyte ratio; PLR, platelet–lymphocyte ratio; ALP, alkaline phosphatase; OIPI, osteosarcoma immune prognostic index.

We developed the LIPI with LDH and dNLR according to a previous study (30). Surprisingly, we found that HBDH was also a significant predictive factor in osteosarcoma with a high AUC value in t-ROC. Thus, we constructed the osteosarcoma immune prognostic index (OIPI) combining LIPI and HBDH (**Figure 1A**). As shown in **Figures 1A**, the t-ROC analysis demonstrated that the AUC of the OIPI was larger than that of inflammatory markers (dNLR, LDH, HBDH, LIPI) and clinical features (gender, age, tumor site, pathological fracture, and IPNs) (**Figures 1A, B**). This result showed that the OIPI performed better in predicting the metastasis than other hematological factors and clinical features. The OIPI divided 184 patients into four groups according to the cutoff values of hematological factors, with the no-OIPI group for 34 patients, light-OIPI group for 35 patients, moderate-OIPI group for 75 patients, and severe-OIPI group for 40 patients (P < 0.0001). For example, a patient with high dNLR, high LDH, and high HBDH was thought to have three high cutoff values and was categorized as severe OIPI (**Figure 2**).

Univariate analysis and multivariate analysis were conducted to further explore the correlation between predictive factors and metastasis in 184 patients. According to the univariate analysis, the pathological fracture (hazard ratio (HR) 2.006; [95% confidence interval CI] 1.018–3.953, P = 0.044), IPNs (HR 2.291 [95% CI] 1.398–3.756, P = 0.001), NLR (HR 2.182 [95% CI] 1.331–3.578, P = 0.002), PLR (HR 2.289 [95% CI] 1.363–3.845, P = 0.002), ALP (HR 2.189 [95% CI] 1.316–3.64, P = 0.003), and OIPI (HR 2.140 [95% CI] 1.590–2.88), P < 0.001) were associated with metastasis and were selected to perform multivariate analysis to identify independent risk factors for metastasis. The multivariate analysis revealed that IPNs (HR 1.717 [95% CI] 1.022–2.887, P = 0.041), PLR (HR 2.185 [95% CI] 1.247–3.829, P = 0.006), and OIPI (HR 1.950 [95% CI] 1.336–2.846, P < 0.001) were independent risk factors for metastasis (**Figures 2B**).

### Effect of OIPI in evaluating the metastatic sites and metastatic time of patients with metachronous metastasis in 184 osteosarcoma patients

We further explored the clinical significance of the OIPI in patients developing metachronous metastasis. Spearman’s correlation analysis was applied to explore the relationship between OIPI and metastasis sites (**Figure 2D**). The results demonstrated that the OIPI was significantly associated with the metastasis sites, in which more patients developed lung metastasis and extrapulmonary metastasis in the high-OIPI group (moderate and severe) than in the low-OIPI group (none and light). The Kruskal–Wallis test was used to compare differences in metachronous metastases in the four OIPI groups (**Figure 2E**). As shown, compared with the no-OIPI group, the moderate-OIPI group and the severe-OIPI group developed metachronous metastases earlier (P = 0.016 and P = 0.017, respectively).

### Effect of the OIPI in predicting metachronous metastasis in patients with IPNs or without IPNs

**Figure 3:**
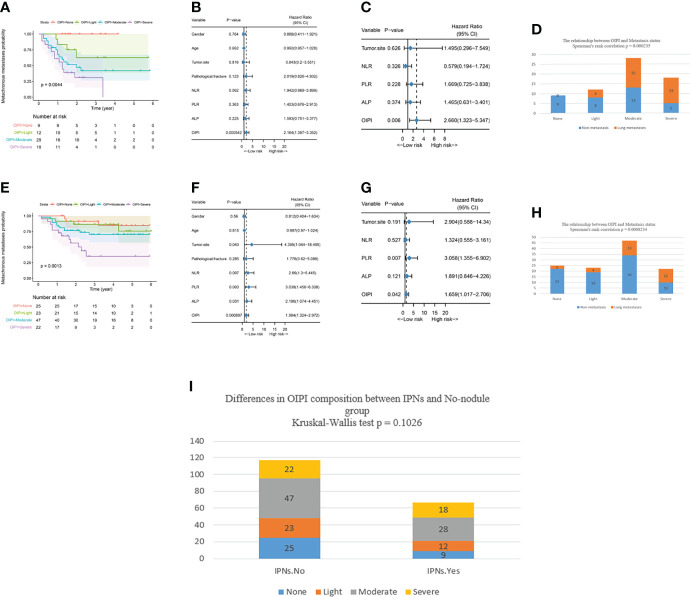
Effect of the OIPI in predicting metachronous metastasis in patients with no nodule or IPNs. **(A)** OIPI divided 67 patients with IPNs into four groups. There are significant differences among patients in the four groups (P = 0.0044). **(B)** Forest plot showing the results of univariate Cox regression analysis of hematological markers and clinical features in 67 patients with IPNs. **(C)** Forest plot showing the results of multivariate Cox regression analysis of hematological markers and clinical features in 67 patients with IPNs. **(D)** Spearman’s rank correlation test demonstrated that the OIPI was related to metastasis site in patients with IPNs (P = 0.000235). **(E)** OIPI divided 117 patients with no nodule into four groups. There were significant differences among patients in the four groups (P = 0.0013) **(F)** Forest plot showing the results of univariate COX regression analysis of hematological markers and clinical features in 117 patients with no nodule. **(G)** Forest plot showing the results of multivariate Cox regression analysis of hematological markers and clinical features in 117 patients with no nodule. **(H)** Spearman’s rank correlation test demonstrated that the OIPI was related to metastasis site in patients with no nodule (P = 0.0008234). **(I)** The Kruskal–Wallis test showed that there was no significant difference in the composition of OIPI between the IPN group and the no-nodule group. OIPI, osteosarcoma immune prognostic index; IPNs, indeterminate pulmonary nodules; NLR, neutrophil–lymphocyte ratio; PLR, platelet–lymphocyte ratio; ALP, alkaline phosphatase.

To evaluate the stability of the OIPI and improve its accurate clinical application, we explored the application of the OIPI in the subgroup with IPNs (36.4%) or no nodule (63.6%). In patients with IPNs, the OIPI divided patients into four groups with the no-OIPI group for 9, light-OIPI group for 12, moderate-OIPI group for 28, and severe-OIPI group for 18 (**Figure 3A**) (P = 0.0044). According to the results of univariate analysis and multivariate analysis, the OIPI was the independent risk factor for metachronous metastasis (**Figures 3B,C**). These results indicated that the OIPI was a stable tool in predicting the metachronous metastasis. In addition, Spearman’s rank correlation test demonstrated that OIPI was related to metastatic sites in patients with IPNs. Patients with IPNs in moderate or severe OIPI groups developed more lung metastases (**Figure 3D**).

In patients with no nodule, the OIPI divided patients into four groups with the no-OIPI group for 25, light-OIPI group for 23, moderate-OIPI group for 47, and severe-OIPI group for 22 (**Figure 3E**) (P = 0.0013). According to the results of the univariate analysis and multivariate analysis, the PLR and OIPI were independent risk factors for metastasis in 117 no-nodule patients (**Figures 3F,G**). Similarly, the Spearman’s rank correlation test demonstrated that OIPI was also related to metastatic sites in patients with no nodule. Patients with no nodule in the moderate- or severe-OIPI groups developed more lung metastases (**Figure 3H**).

We also applied the Kruskal–Wallis test to analyze the difference in the composition of OIPI between the IPN group and the no-nodule group. The result shows that there is no significant difference in the composition of OIPI between the IPN group and the no-nodule group (**Figure 3**).

### Effect of the combination of the OIPI and IPNs in predicting the MFS and metastatic time

**Figure 4:**
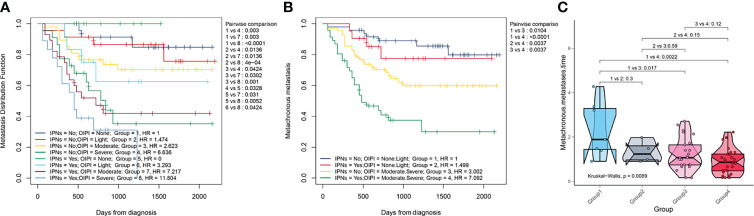
Effect of the combination of the OIPI and IPNs in predicting the MFS and metastatic time. **(A)** A total of 184 osteosarcoma patients were divided into eight groups according to the combination of OIPI and IPNs. The difference in metastatic predictive ability among eight groups was shown. **(B)** A total of 184 osteosarcoma patients were divided into four groups according to the combination of OIPI and IPNs. The difference in metastatic predictive ability among the four groups was shown. **(C)** The Kruskal–Wallis test demonstrated that there was a difference in metastatic time among the four groups (P = 0.0089). Patients in the moderate- and severe-OIPI groups developed metachronous metastasis earlier than those in the no-OIPI group (P = 0.017 and P = 0.0022, respectively). OIPI, osteosarcoma immune prognostic index; IPNs, indeterminate pulmonary nodules; MFS, metastasis-free survival.

We also explored the metastatic predictive function of the combination of the OIPI and IPNs in osteosarcoma patients. A total of 184 osteosarcoma patients were divided into eight groups according to the initial CT report (no-nodule or IPNs) and the OIPI classification. As shown in **Figure 4A**, in patients without nodules, group 4 had a higher probability of metastasis than group 1 (P = 0.003), group 2 (P = 0.0136), or group 3 (P = 0.0424). Among 184 osteosarcoma patients, patients in group 7 and group 8 were more likely to develop metastasis than patients in the other groups (group 1 vs. group 7, P = 0.003; group 1 vs. group 8, P < 0.0001; group 2 vs. group 7, P = 0.0136; group 2 vs. group 8, P < 0.0001; group 3 vs. group 7, P = 0.0302; group 3 vs. group 8, P = 0.001; group 5 vs. group 7, P = 0.031; group 5 vs. group 8, P = 0.0052; group 6 vs. group 8, P = 0.0424). Therefore, the patients in group 4, group 7, and group 8 were considered the metastasis high-risk groups. Then, we divided the patients into four groups according to the presence of IPNs (yes vs. not) and the OIPI (none and light (low OIPI) vs. moderate and severe (high OIPI)). As shown in **Figures 4B,C**, patients in group 3 and group 4 were the metastasis high-risk groups and always developed metastasis earlier than patients in group 1 (P = 0.017 and P = 0.0022, respectively).

### Construction and validation of the OIPI-based nomogram

**Figure 5:**
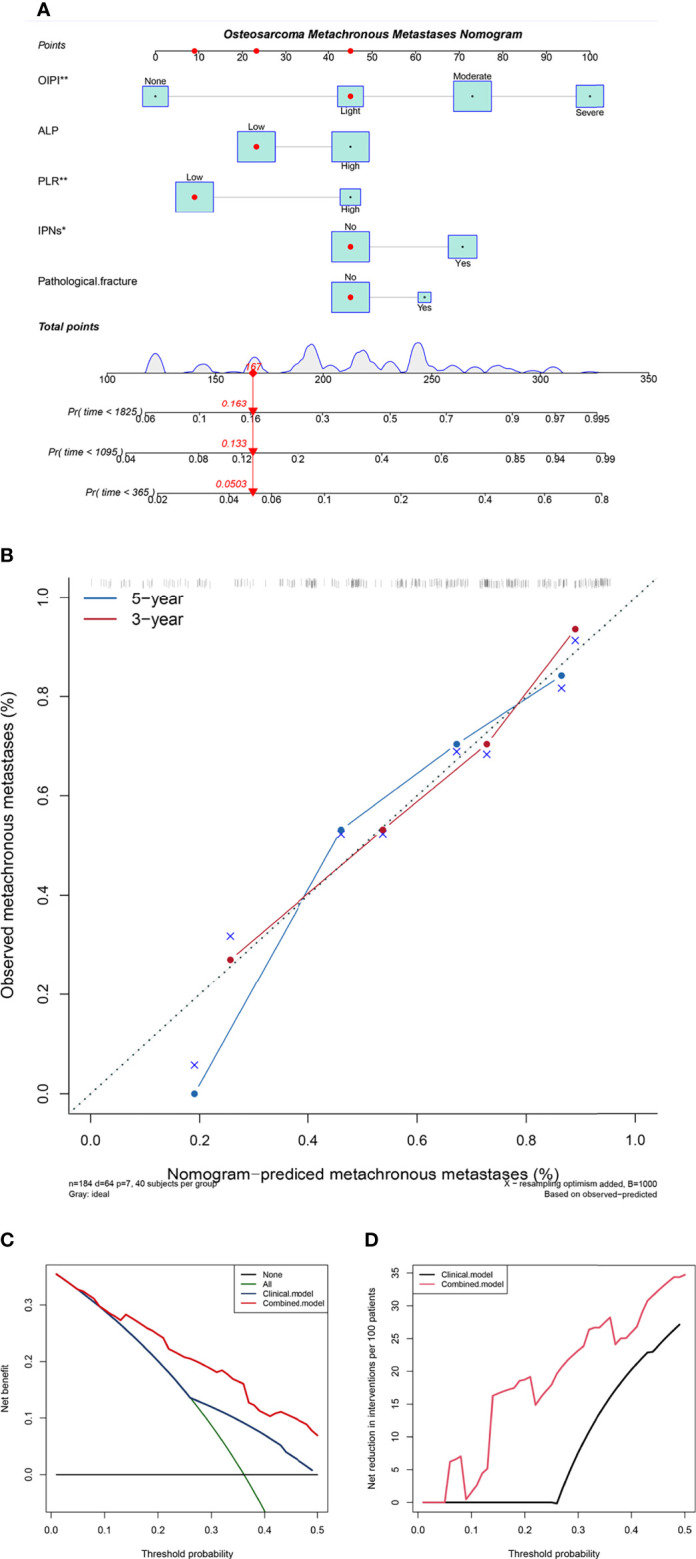
Construction and validation of the osteosarcoma metachronous metastasis nomogram. **(A)** The nomogram was constructed by combining OIPI, ALP, PLR, IPNs, and pathological fracture, and the sum of the scores for each covariate was the nomogram total score. **(B)** Calibration curves for the nomogram predicting 3- and 5-year metachronous metastasis of osteosarcoma patients. **(C)** The clinical net benefit curve of this nomogram. **(D)** Clinical net reduction curve for the nomogram. OIPI, osteosarcoma immune prognostic index; ALP, alkaline phosphatase; PLR, platelet–lymphocyte ratio; IPNs, indeterminate pulmonary nodules; *P < 0.05, **P < 0.01, ***P < 0.001.

To improve the clinical application of the OIPI, we constructed a nomogram combining the OIPI with clinical features. As shown in **Figure 5A**, Cox proportional hazards regression assigned a score based on the hazard ratio for each covariate, and the sum of the scores for each covariate was the nomogram total score. The C-index of this osteosarcoma metachronous metastasis nomogram was 0.72, and the calibration curve indicated that this nomogram could accurately predict 3- and 5-year OS (**Figure 5B**). Eventually, we also explored the clinical benefits of this nomogram with clinical DCA (**Figures 5C,D**). Our results demonstrate that the addition of this nomogram with the OIPI could bring significant net benefits over the model with only clinical features.

## Discussion

In this study, we developed an osteosarcoma immune prognostic index (OIPI) for osteosarcoma patients with the combination of LDH, dNLR, and HBDH. The OIPI stratifies patients into four groups, none, light, moderate, and severe. The OIPI performs better in metastatic predictive ability than other hematological parameters and clinical features. Meanwhile, our results indicate that the OIPI is a stable predictive tool and could be used to evaluate the metastasis sites and MFS. Notably, this predictive model may help identify patients who develop early metachronous metastasis. Additionally, we also explored the predictive power of the combination of OIPI and IPNs; our results demonstrated that the OIPI could help clarify the nature of IPNs in chest CT. Also, the combination of OIPI and IPNs could help identify metastatic high-risk patients. Furthermore, we constructed a nomogram that had good predictive accuracy in predicting 3- and 5-year overall survival and is susceptible to bring significant net benefits to osteosarcoma patients.

Almost all patients with osteosarcoma have micrometastases at initial diagnosis, but only one-fifth of patients can be detected as metastasis status (3). In recent years, many efforts have been made to improve the prognosis of metastatic patients, such as the advancement of immune checkpoint inhibitors or TKI agents (34, 35). Unfortunately, several recent clinical trials have shown limited clinical benefit of these drugs for osteosarcoma (35, 36). Therefore, more focus on identifying micrometastases or early diagnosis of metastatic status may be an effective strategy. The lung is the most common site of osteosarcoma metastasis, and lung metastasis is associated with poor prognosis with a 5-year survival rate of 30%–40% (37–39).With the continuous development of CT detection technology, IPNs are detected in more osteosarcoma patients (16, 17, 40, 41), whereas there is still no consensus on the clinical relevance of IPNs and metastasis; it is currently based on nature (such as mineralization, round, and size greater than 5 mm) on CT alone to determine which IPNs will progress to lung metastases (13–15). Therefore, in clinical work, regular reexamination of CT is usually recommended for these patients. It is difficult to give personalized diagnosis and treatment opinions, meaning that patients with IPNs may miss the best time for treatment (11, 12, 16). In addition, although needle biopsy, thoracoscopy, and exploratory thoracotomy are considered to be options for defining the nature of nodules, the heterogeneity of IPNs renders their use limited due to inaccessibility to deep metastases, micrometastases, and invasiveness (11, 18). Therefore, this study expects to develop a predictive tool to assist in CT detection and make a personalized treatment plan for patients with IPNs, rather than recommending regular CT reviews to all patients.

An ideal prediction tool must be available, repeatable, predictable, and cost-effective. Recently, several novel assays such as lncRNAs have been developed. However, these methods are still some ways from clinical applications due to their high cost, difficulty in being obtained, and unified detection methods (42–45). Meanwhile, the predictive effect of hematological markers has been widely explored. A large number of hematological markers have been reported to have great predictive potential in osteosarcoma patients (23–28). Hematological markers could be ideal prediction tools because they are accessible to obtain and are reproducible. The LIPI is a novel hematological marker introduced by Mezquita et al., which is important for immunotherapeutic options and long-term survival prediction in advanced lung cancer and extrapulmonary cancer (30, 32, 33). The LIPI is composed of dNLR and LDH. Compared with the dNLR and LDH alone, the LIPI could better represent the inflammatory status and predict the prognosis of cancer patients (31). At present, the predictive role of the LIPI in osteosarcoma patients has not been reported. Given the important role of the LIPI in predicting prognosis and guiding immunotherapy selection, we hypothesized that the LIPI also has predictive potential of metastasis in osteosarcoma patients. Our results verified this hypothesis. In 184 osteosarcoma patients, the LIPI showed a better predictive ability of metastasis than dNLR or LDH alone (**Figure 1A**). At the same time, the results also showed that HBDH, an isoenzyme of LDH, had prognostic significance. The predictive ability of HBDH in predicting metastasis was even comparable to that of LIPI in the first 4 years after diagnosis (**Figure 1A**). Therefore, we combined the LIPI and HBDH to create the OIPI. The results showed that the predictive power of the OIPI was higher than that of other hematological markers and clinical natures (**Figures 1A,B**). Surprisingly, the predictive ability of the OIPI was even higher than that of the LIPI, implying that the OIPI may be more suitable for predicting metastasis in osteosarcoma patients. The possible reason is that the inclusion of HBDH promotes the OIPI to be a comprehensive inflammatory indicator, which helps the OIPI more fully reflect the inflammatory status and correlate with the metastatic status. On the other hand, the combination of dNLR, LDH, and HBDH can reduce potential bias because each individual index may be affected by various factors.

Excitingly, our results also revealed that the OIPI could predict metastatic sites and metastatic time. Patients in the moderate- and severe-OIPI groups were more likely to develop metachronous metastases, including lung and extra-lung metastases (**Figure 2E**). Therefore, for patients in the moderate- and severe-OIPI groups, both a more frequent chest CT and regular bone imaging or positron emission tomography-computed tomography (PET-CT) are recommended to timely detect extra-lung metastasis. (**Figure 2D**). In addition, patients in the severe-OIPI group may develop metastasis earlier than those in the no-OIPI group (**Figure 2E**). The reason may be that patients in the severe-OIPI group had a more severe inflammatory response, activation of pro-EMT signaling pathways, close interaction between immune cells and tumor cells, and more increased CTC concentrations. In that situation, tumor cells were more likely to colonize distant sites to form metastases (46, 47).

Considering the complexity of the tumor metastasis process, it is difficult to imagine that there is a test method that can independently and accurately predict tumor metastasis. Therefore, it is more important to combine OIPI with existing clinical features such as imaging features to more accurately predict metastasis in patients. Our results suggested that the OIPI can be used as a complementary tool for chest CT to synergistically evaluate the nature of IPNs. As shown in **Figure 3**, in both patients with IPNs and without nodules, the OIPI could evenly divide patients into four groups and was an independent predictor of metachronous metastasis, suggesting that the OIPI is a stable predictive tool. In addition, OIPI was associated with the metastatic site in patients with IPNs. In patients with IPNs, patients with a high OIPI had a higher probability to develop lung metastasis, revealing that the OIPI could assist in determining the nature of IPNs (**Figure 3D**). Furthermore, to investigate the predictive ability of metastasis in the OIPI combined with IPNs, we divided patients into different risk groups according to the OIPI and IPNs. In the high-risk group (moderate and severe OIPI groups), patients with IPNs had a higher probability of developing metastasis (**Figures 4A,B**) (group 7 vs. group 1, HR = 7.217, P = 0.003; group 8 vs. group 1, HR = 11.804, P < 0.0001). In addition, the combination of OIPI and IPNs also had some advantages in assessing the metastatic time. As shown, group 4 had an earlier onset of metastases than group 1 (P < 0.0001). As a result, we recommend that more attention should be paid to patients in the moderate- and severe-OIPI groups with IPNs. In these high-risk patients, long-term close follow-up, more frequent chest CT, or invasive surgery to identify metastases may be helpful for the early diagnosis of metastatic lesions. On the other hand, for patients in the no-OIPI group, the probability of malignant transformation of IPNs was low; thus, long-term follow-up and regular chest CT are necessary to avoid unnecessary invasive procedures (**Figures 4A,B**). Simply put, the OIPI can be used as a complementary tool of IPNs for predicting metastasis in clinical practice. For patients with IPNs, clinicians could combine the OIPI and chest CT to individualize the risk classification. Moreover, clinicians could develop individual treatment plans and offer accurate patient consultations.

Furthermore, we also constructed an OIPI-based nomogram for predicting the metachronous metastasis in osteosarcoma. A nomogram is an accurate and convenient mathematical model that can predict specific endpoints. It can help clinicians better diagnose and determine treatment options. Applying nomograms to assess metastatic status is necessary (48). Nomograms are rarely reported in predicting metastasis in osteosarcoma. Most of the nomograms are established by integrating clinical parameters and genes associated with metastasis (48–51). However, due to the imprecision of clinical features and the clinical inapplicability of genes, the clinical application of these nomograms was limited. In the current study, we included the indicators significantly associated with metachronous metastasis including OIPI, initial CT report, and PLR in the construction of the nomogram (**Figure 5A**). At the same time, we also included ALP, pathological fracture, and other indicators considering their important prognostic role in osteosarcoma. According to the patients’ individual information and corresponding value, we can obtain a total score to predict the risk of metachronous metastasis. Our results revealed that this OIPI-based nomogram can accurately predict the 3- and 5-year metastasis rates of osteosarcoma patients (**Figure 5B**). Additionally, compared with the prediction model without OIPI, the OIPI-based nomogram can benefit more osteosarcoma patients in the diagnosis and treatment process (**Figures 5C,D**). Based on the lack of metastasis prediction tools in clinical practice, we believe that this nomogram could be a good prediction tool for metachronous metastasis in osteosarcoma patients.

The mechanism of the OIPI in predicting metastasis may because it can reflect the extent of the inflammatory response and evaluate the metastasis status. As is well known, metastasis originates from the invasion of cancer cells from the epithelial layer to the surrounding tissue and the acquisition of the EMT (epithelial to mesenchymal transition) phenotype. Then the cancer cells could break through the basement membrane, invade the tissue, and reach lymphatic vessels or blood vessels for further spread (46, 47). Inflammation affects cancer invasion, EMT, and cell migration at various levels (52). Cytokines, tumor-specific inflammatory signals, and immune cells promote the migration of tumor cells and the establishment of a metastatic microenvironment by interacting with cancer cells or the microenvironment (52). Inflammatory cells such as monocytes and neutrophils can further aid in adhesion and extravasation processes, which will accelerate the adhesion of metastatic seeds, form complexes with cancer cells, regulate the adhesion and translocation throughout the vessel wall, and finally establish and maintain a metastatic niche (53–55). Oligocellular complexes between cancer cells themselves or immune cells–cancer cells also help to protect these metastatic seeds from immune surveillance (56). Platelets protect CTCs from lethal attack by the immune system or other pro-apoptotic stimuli and provide signals to establish a pro-metastatic niche environment that ultimately promotes tumor growth and metastasis (57). LDH, as a classical prognostic predictor of osteosarcoma, often detects higher LDH levels in metastatic patients than in non-metastatic patients (58, 59).At the same time, some clinical studies have also shown that an increase in lactate concentration is also associated with the subsequent development of nodules or distant metastasis (60). This shows the value of detecting LDH levels in predicting metastasis. As the cornerstone of OIPI, dNLR, LDH, and HBDH are all associated with metastasis. The constructed OIPI may reflect the extent of inflammation affecting cancer cell invasion, EMT, and cell migration at different levels, thereby assessing the possibility of patient metastasis.

It must be acknowledged that our study has several limitations. First, this single-center and retrospective study may have resulted in selection bias. Second, the results of the relationship between OIPI and metastatic and metastasis sites need to be interpreted with caution. Since there were only three cases of metastasis in the no-OIPI group, this may lead to instability in exploring the relationship between the OIPI and metastatic time. In the next stage, we plan to conduct further studies with larger numbers of participants and longer follow-up to further verify the clinical significance of the OIPI in predicting the metastatic time. Finally, the prognostic value of HBDH in osteosarcoma still needs further validation. In this study, we preliminarily investigated the prognostic value of HBDH, the isozyme of LDH. Surprisingly, HBDH behaved comparably to LDH in our cohort. However, studies on the prognostic value of HBDH in cancer patients are very scarce. In osteosarcoma, only our study reported the prognostic value of HBDH. Therefore, further studies are needed to elucidate the predictive ability of HBDH in patients with osteosarcoma or even cancer patients. Despite the limitations, our study is the first to interrogate the clinical significance of hematological markers with IPNs, providing a new idea for subsequent studies. Further validation of the OIPI in relation to metastatic time and treatment effect is needed. It is even more important to explore whether the OIPI can truly produce a net clinical benefit. For example, for patients with a high OIPI, whether closer follow-up and more frequent chest CT can benefit patients or not needs to be further determined.

## Conclusion

In conclusion, this study is the first to assist chest CT in diagnosing the nature of IPNs in osteosarcoma based on hematological markers. Our findings suggest that an OIPI with a LIPI is superior to other hematological markers, and the OIPI can be used as an auxiliary tool to determine the malignant transformation tendency of IPNs. The combination of the OIPI with IPNs can further improve the metastatic predictive ability in osteosarcoma patients. Further studies are needed to validate our findings.

## Data availability statement

The original contributions presented in the study are included in the article/**Supplementary material**. Further inquiries can be directed to the corresponding authors.

## Ethics statement

The studies involving human participants were reviewed and approved by the Ethics Committee of West China Hospital, Sichuan University. Written informed consent to participate in this study was provided by the participants’ legal guardian/next of kin.

## Author contributions

XH, ML, and CT designed the research study. ML and LL performed the research. CZ provided help and advice on revising the manuscript. YL analyzed the data. XH and LM wrote the manuscript. All authors contributed to editorial changes in the manuscript. All authors read and approved the final manuscript.

## Funding

This study is funded by the Science and Technology Research Program of Sichuan Province (2020YFS0036), and 1·3·5 project for disciplines of excellence, West China Hospital, Sichuan University (ZYJC18036).

## Conflict of interest

The authors declare that the research was conducted in the absence of any commercial or financial relationships that could be construed as a potential conflict of interest.

## Publisher’s note

All claims expressed in this article are solely those of the authors and do not necessarily represent those of their affiliated organizations, or those of the publisher, the editors and the reviewers. Any product that may be evaluated in this article, or claim that may be made by its manufacturer, is not guaranteed or endorsed by the publisher.
