# Supplementary material for: Osteosarcoma immune prognostic index can indicate the nature of indeterminate pulmonary nodules and predict the metachronous metastasis in osteosarcoma patients
Source: Front Oncol. 2022 Jul 22;12:952228. doi: 10.3389/fonc.2022.952228 (PMC9354693; doi:10.3389/fonc.2022.952228)
Supplement: Supplementary file 1 [file Table_1.docx]

Supplementary Table 1 Optimal cutoff values for hematological markers

| Hematological Markers | AUC | Cutoff Values |
| --- | --- | --- |
| NLR | 0.594 | 2.77 |
| PLR | 0.569 | 227.14 |
| LMR | 0.519 | 5.02 |
| dNLR | 0.607 | 2.05 |
| LDH (IU/L) | 0.590 | 158.00 |
| HBDH (IU/L) | 0.680 | 126.00 |
| CK (IU/L) | 0.537 | 215.00 |
| ALP (IU/L) | 0.535 | 91.00 |

AUC, area under curve in the Receiver Operating Characteristic Curve; NLR, neutrophil–lymphocyte ratio; PLR, platelet–lymphocyte ratio; LMR, lymphocyte-monocyte ratio; LDH, lactate dehydrogenase; dNLR, derived neutrophil to lymphocyte ratio; HBDH, hydroxybutyrate dehydrogenase; CK, creatine kinase; ALP, alkaline phosphatase;
